# Supplementary material for: Three stepwise pH progressions in stratum corneum for homeostatic maintenance of the skin
Source: Nat Commun. 2024 May 15;15:4062. doi: 10.1038/s41467-024-48226-z (PMC11096370; doi:10.1038/s41467-024-48226-z)
Supplement: Supplementary file 1 — Supplemenatary Information [file 41467_2024_48226_MOESM1_ESM.pdf]

Supplementary Information

Three stepwise pH progressions in stratum corneum for homeostatic maintenance of the skin

Keitaro Fukuda[1,2,8], Yoshihiro Ito[2,8], Yuki Furuichi[1,2,8], Takeshi Matsui[1,3], Hiroto Horikawa[1,2] Takuya Miyano[4], Takaharu Okada[5,6], Mark van Logtestijn[4], Reiko J Tanaka[4], Atsushi Miyawaki[7], and Masayuki Amagai[1,2,*]

[1]Laboratory for Skin Homeostasis, RIKEN Center for Integrative Medical Sciences, Kanagawa, Japan

[2]Department of Dermatology, Keio University School of Medicine, Tokyo, Japan.

[3]Laboratory for Evolutionary Cell Biology of the Skin, School of Bioscience and Biotechnology, Tokyo University of Technology, Tokyo, Japan

[4]Department of Bioengineering, Imperial College London, London, United Kingdom.

[5]Laboratory for Tissue Dynamics, RIKEN Center for Integrative Medical Sciences, Kanagawa, Japan

[6]Graduate School of Medical Life Science, Yokohama City University, Kanagawa, Japan

[7]Laboratory for Cell Function Dynamics, RIKEN Center for Brain Science, Saitama, Japan.

[8]These authors contributed equally: Keitaro Fukuda, Yoshihiro Ito, and Yuki Furuichi.

*Correspondence should be addressed to: amagai@keio.jp

Fig. S1

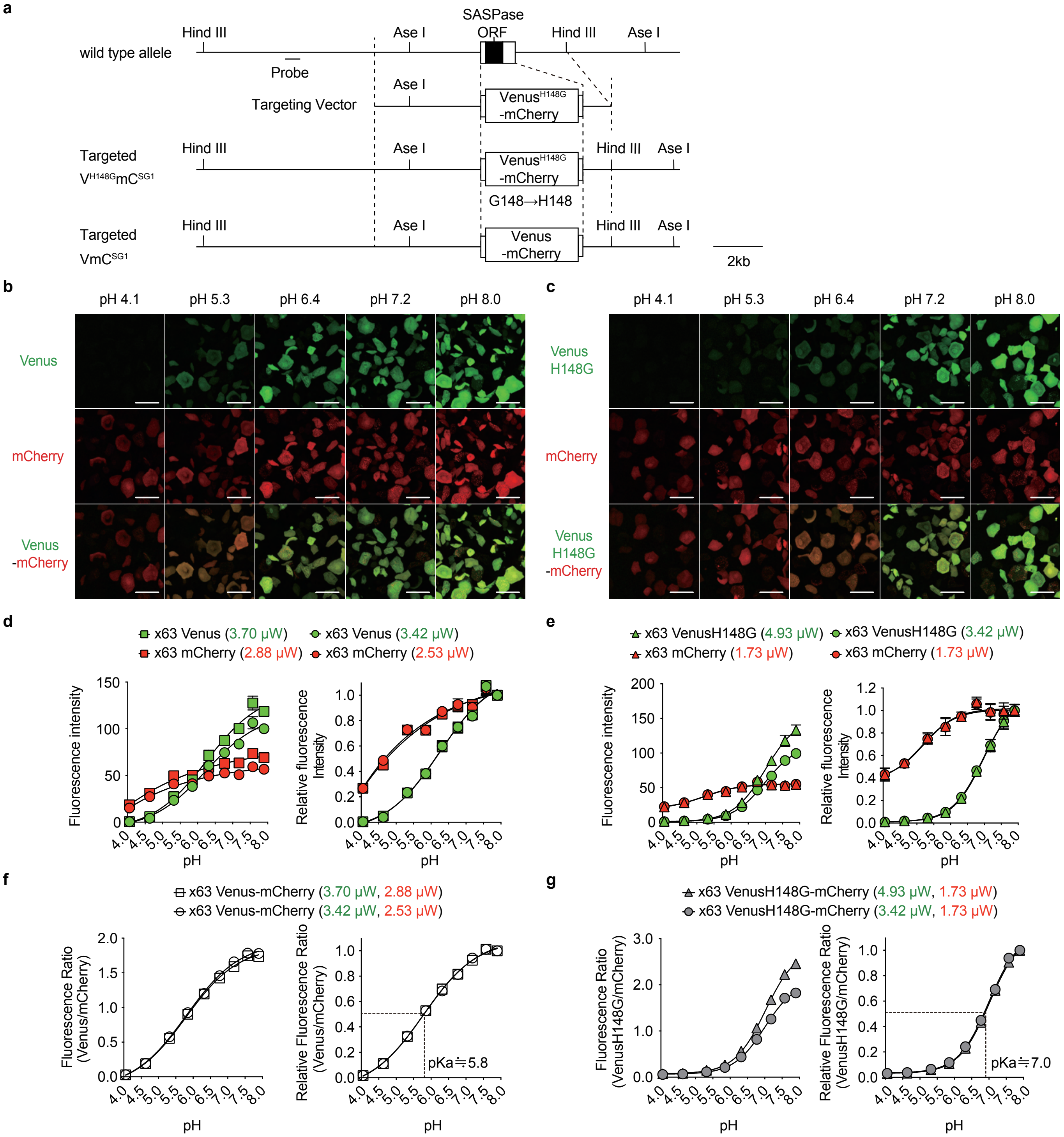

a
SASPase ORF
Hind III
Ase I
wild type allele
Probe
Targeting Vector
Venus H148G -mCherry
Targeted V H148G mC SG1
G148→H148
Targeted VmC SG1
Venus -mCherry
2kb
b
c
pH 4.1
pH 5.3
pH 6.4
pH 7.2
pH 8.0
Venus
mCherry
Venus -mCherry
Venus H148G
Venus H148G -mCherry
d
x63 Venus (3.70 μW)
x63 Venus (3.42 μW)
x63 mCherry (2.88 μW)
x63 mCherry (2.53 μW)
Fluorescence intensity
Relative fluorescence Intensity
pH
e
x63 VenusH148G (4.93 μW)
x63 VenusH148G (3.42 μW)
x63 mCherry (1.73 μW)
x63 mCherry (1.73 μW)
f
x63 Venus-mCherry (3.70 μW, 2.88 μW)
x63 Venus-mCherry (3.42 μW, 2.53 μW)
Fluorescence Ratio (Venus/mCherry)
Relative Fluorescence Ratio (Venus/mCherry)
pKa≒5.8
g
x63 VenusH148G-mCherry (4.93 μW, 1.73 μW)
x63 VenusH148G-mCherry (3.42 μW, 1.73 μW)
Fluorescence Ratio (VenusH148G/mCherry)
Relative Fluorescence Ratio (VenusH148G/mCherry)
pKa≒7.0

Supplementary Figure 1. Evaluation of pH indicators Venus−mCherry and VenusH148G-mCherry

a Schematic of the strategy used to generate mice expressing Venus-mCherry and VenusH148G-mCherry from SG1 cells. Representative confocal microscopy images of (**b**) VmC^{SG1}-derived and (**c**) $VH148GmC^{SG1}$-derived SG1 cells in various pH buffers. Scale bar, 50 µm. Data are representative of at least three independent experiments (**b** and **c**). **d** and **e** Fluorescence intensity (left) and relative fluorescence intensity (right) of (**d**) Venus and mCherry and (**e**) VenusH148G and mCherry proteins expressed in isolated SG1 cells under two laser powers, as indicated. **f** and **g** Fluorescence ratio (left) and relative fluorescence ratio (right) of (**f**) Venus to mCherry and (**g**) VenusH148G to mCherry expression in isolated SG1 cells under two laser powers. Data are shown as mean ± SEM and have been pooled from three experiments totaling 30 cells from three biologically independent animals, respectively, and were fitted using a sigmoidal nonlinear regression model (**d**-**g**). Source data are provided as a Source Data file.

Fig. S2

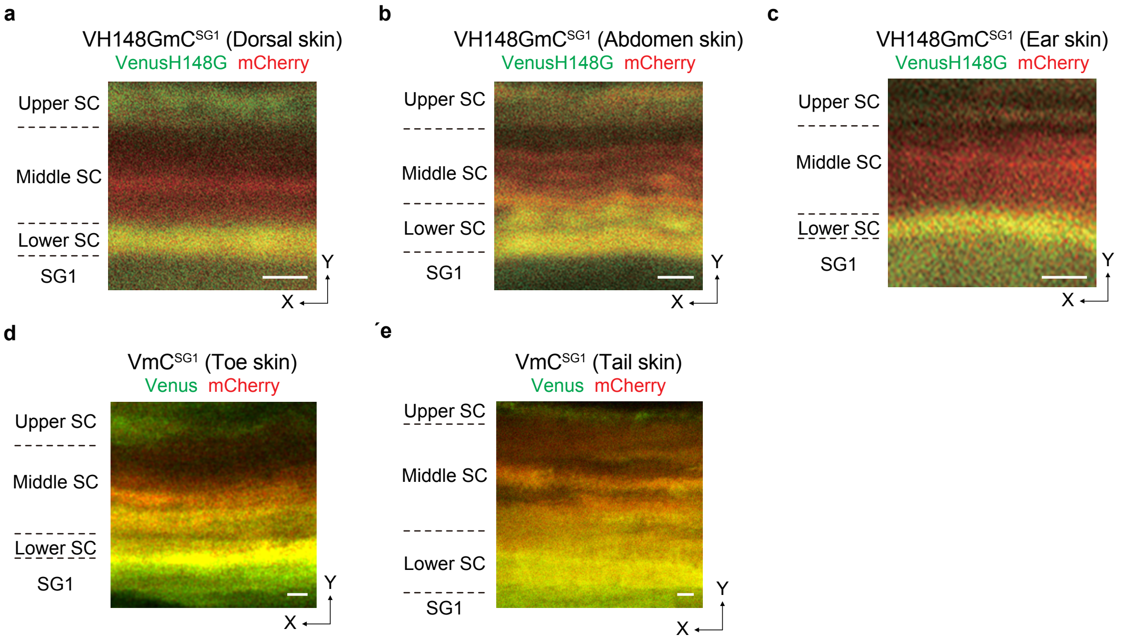

a
VH148GmC^{SG1} (Dorsal skin)
VenusH148G mCherry
Upper SC
Middle SC
Lower SC
SG1
Y
X
b
VH148GmC^{SG1} (Abdomen skin)
VenusH148G mCherry
Upper SC
Middle SC
Lower SC
SG1
Y
X
c
VH148GmC^{SG1} (Ear skin)
VenusH148G mCherry
Upper SC
Middle SC
Lower SC
SG1
Y
X
d
VmC^{SG1} (Toe skin)
Venus mCherry
Upper SC
Middle SC
Lower SC
SG1
Y
X
´e
VmC^{SG1} (Tail skin)
Venus mCherry
Upper SC
Middle SC
Lower SC
SG1
Y
X

Supplementary Figure 2. Visualization of SC-pH using VH148GmC^{SG1} and VmC^{SG1} mice

a–**c** High-magnification X-Y plane confocal images of SC-pH zones of the (**a**) dorsal, (**b**) abdominal, and (**c**) ear skin of VH148GmC^{SG1} mice. Scale bar, 1 μm. **d** and **e** High-magnification X-Y plane confocal images of SC-pH zones of the (**d**) toe and (**e**) tail skin of VmC^{SG1} mice. Scale bar, 2 μm. Data are representative of at least three independent experiments (**a**-**e**).

Fig. S3

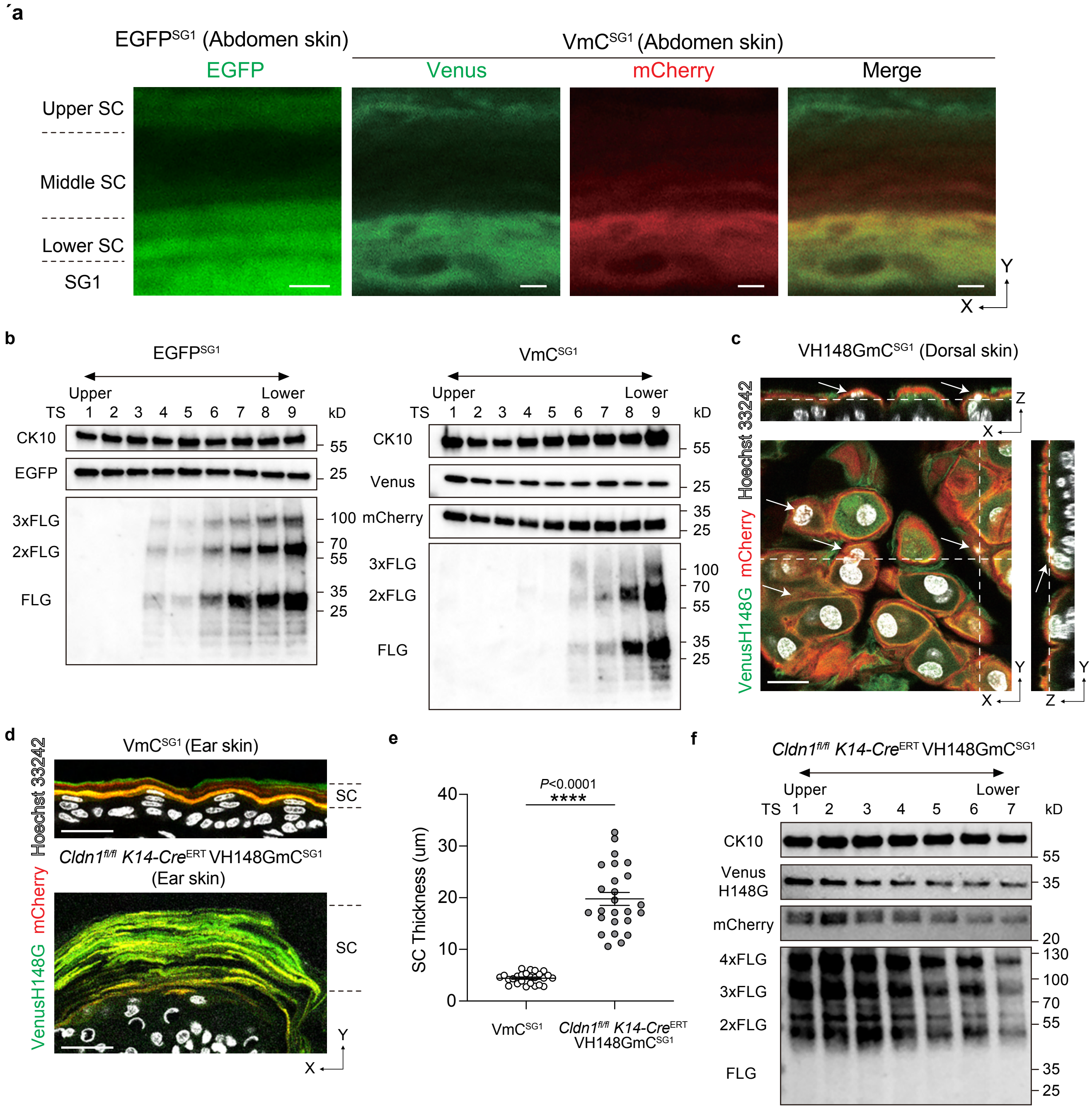
a
EGFPSG1 (Abdomen skin)
VmCSG1 (Abdomen skin)
EGFP
Venus
mCherry
Merge
Upper SC
Middle SC
Lower SC
SG1
b
EGFPSG1
VmCSG1
Upper
Lower
TS
kD
CK10
EGFP
3xFLG
2xFLG
FLG
Venus
mCherry
c
VH148GmCSG1 (Dorsal skin)
VenusH148G mCherry Hoechst 33242
d
VmCSG1 (Ear skin)
Cldn1fl/fl K14-CreERT VH148GmCSG1 (Ear skin)
SC
e
P<0.0001
****
SC Thickness (um)
VmCSG1
Cldn1fl/fl K14-CreERT VH148GmCSG1
f
Cldn1fl/fl K14-CreERT VH148GmCSG1
Venus H148G
4xFLG

Supplementary Figure 3. Visualization of SC-pH using EGFP^{SG1}, VmC^{SG1}, and B6.*Cldn1$^{fl/fl}$ K14*-CreERT VmC^{SG1} mice

a High-magnification X-Y plane confocal images of SC-pH zones of the abdominal skin of EGFP^{SG1} and VmC^{SG1} mice. Merged images of Venus (green) and mCherry (red) expression are shown. Scale bar, 1 µm. **b** Immunoblotting for EGFP, Venus, mCherry, CK10, and filaggrin (FLG) in lysates obtained from tape stripping (TS) samples of EGFP^{SG1} (right) and VmC ^{SG1} (left) mice. **c** Low-magnification X-Y plane and Z-sliced reconstituted representative confocal images of the dorsal skin of VH148GGmC^{SG1} mice injected with Hoechst 33242 (white). White arrows represent SG1 cells with yellow cytoplasm (corneoptosis phase II SG1 cells). Scale bar, 20 µm. **d** Immunofluorescence microscopy of ear skin from VmC^{SG1} and B6.*Cldn1$^{fl/fl}$ K14*-CreERT VmC^{SG1} mice to visualized Venus and mCherry. Scale bar, 10 µm. **e** Thickness of the SC in VmC^{SG1} and B6.*Cldn1$^{fl/fl}$ K14*-CreERT VmC^{SG1} mice ear skin (n=25 spots from three biologically independent animals, each). **f** Immunoblotting for Venus, mCherry, CK10, and FLG in lysates obtained from tape stripping (TS) samples of B6.*Cldn1$^{fl/fl}$ K14*-CreERT VmC^{SG1} mice. Data are shown as mean ± SEM and are representative of at least three independent experiments (**a**–**f**). Two-sided Mann–Whitney's test (**e**). Source data are provided as a Source Data file.

Fig. S4

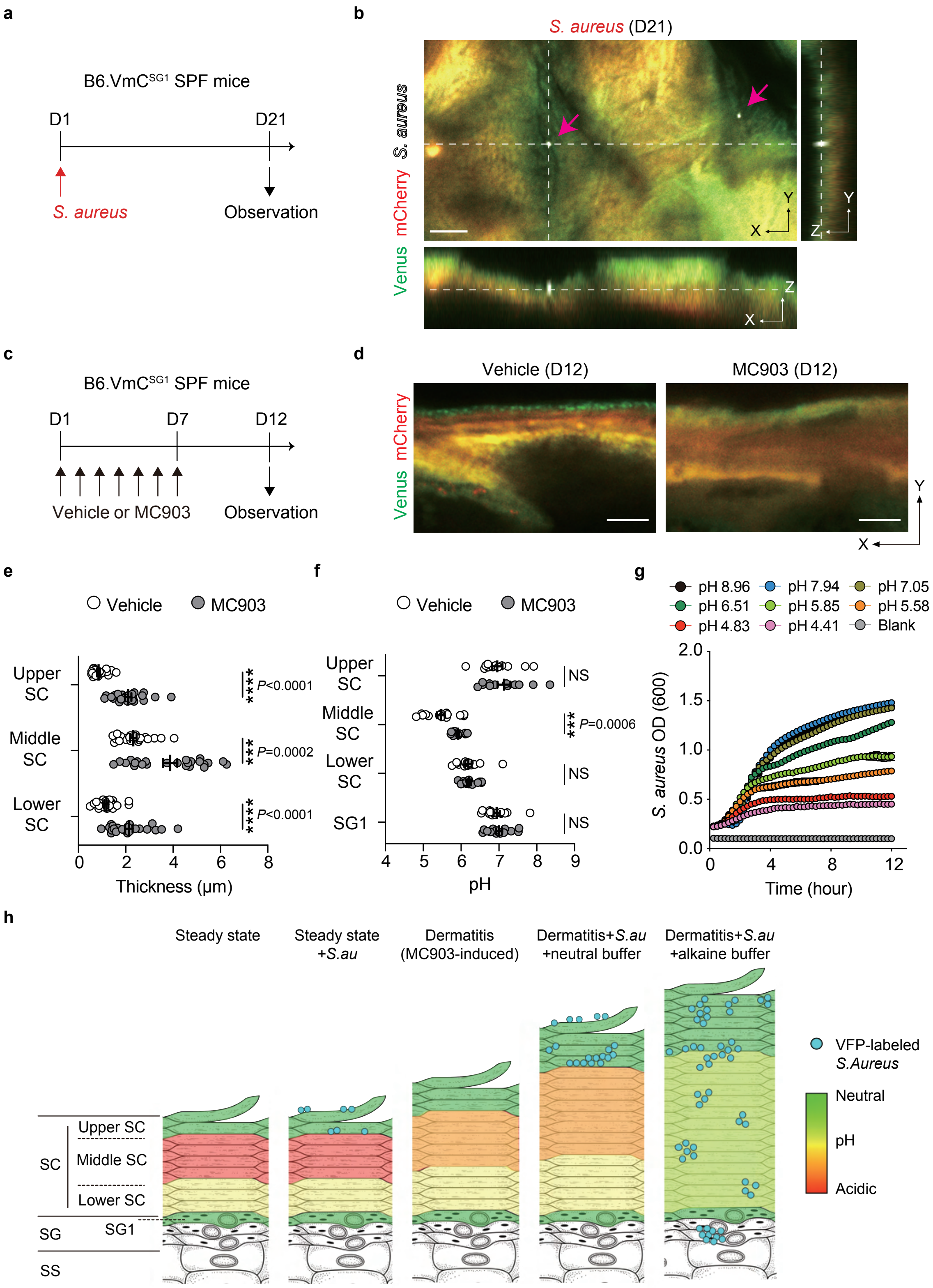

a
B6.VmC^SG1 SPF mice
D1
D21
S. aureus
Observation
b
S. aureus (D21)
Venus mCherry S. aureus
c
B6.VmC^SG1 SPF mice
D1
D7
D12
Vehicle or MC903
Observation
d
Vehicle (D12)
MC903 (D12)
Venus mCherry
e
Vehicle
MC903
Upper SC
Middle SC
Lower SC
P<0.0001
P=0.0002
P<0.0001
Thickness (μm)
f
Vehicle
MC903
Upper SC
Middle SC
Lower SC
SG1
NS
P=0.0006
NS
NS
pH
g
pH 8.96
pH 7.94
pH 7.05
pH 6.51
pH 5.85
pH 5.58
pH 4.83
pH 4.41
Blank
S. aureus OD (600)
Time (hour)
h
Steady state
Steady state +S.au
Dermatitis (MC903-induced)
Dermatitis+S.au +neutral buffer
Dermatitis+S.au +alkaine buffer
Upper SC
Middle SC
Lower SC
SC
SG
SG1
SS
VFP-labeled S.Aureus
Neutral
pH
Acidic

Supplementary Figure 4. Visualization of colonized *S. aureus* in the SC and measurement of SC-pH in inflamed skin

a and **b** B6.VmC^{SG1} mice were inoculated with *S. aureus* on day 1. Mice were analyzed on day 21. **a** Experimental design. **b** Low-magnification X-Y plane and Z-sliced reconstituted representative confocal images of mice colonized with *S. aureus*. Pink arrows represent *S. aureus* signals. Scale bar, 5 µm. **c**–**f** MC903 was applied to B6.VmC^{SG1} mice daily for 7 days (days 1–7) to induce atopic dermatitis-like disease. Mice were analyzed on day 12. **c** Therapy regimen. **d** Representative high-magnification X-Y plane confocal images of mice treated with either vehicle or MC903. Scale bar, 5 µm. **e** Thickness of the upper, middle, and lower SC of B6.VmC^{SG1} mice treated with either vehicle (n=23 spots from three biologically independent animals, each) or MC903 (n=24 spots from three biologically independent animals, each). **f** pH in the each SC-pH zone, and the SG1 of B6.VmC^{SG1} mice treated with vehicle (n=13, 15, 15, and 15 spots from three biologically independent animals, respectively) or MC903 (n=11, 15, 16, and 14 spots from three biologically independent animals, respectively). **g** *S. aureus* growth curves in the pH-adjusted medium. **h** Schematic of VFP-labeled *S. aureus* localization in the SC under various conditions. Data are shown as mean ± SEM and are pooled from three experiments (**e** and **f**) or are representative of at least three independent experiments (**b**, **d**, **g**). ***p < 0.001, ****p < 0.0001; NS, not significant, two-sided Mann–Whitney's test (**e**, **f**). Source data are provided as a Source Data file.

Fig. S5

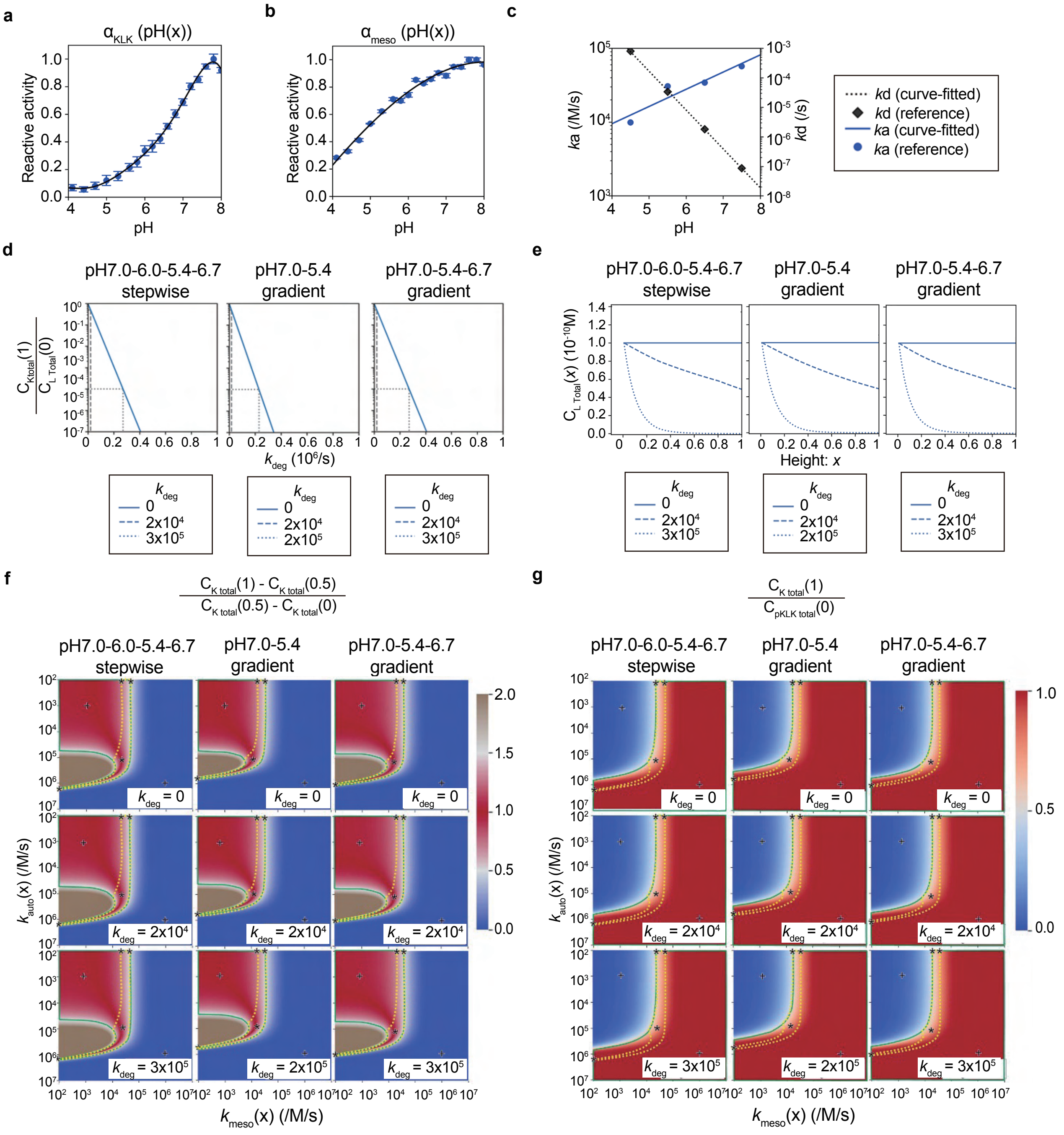
a
αKLK (pH(x))
Reactive activity
pH
b
αmeso (pH(x))
Reactive activity
pH
c
ka (/M/s)
kd (/s)
pH
kd (curve-fitted)
kd (reference)
ka (curve-fitted)
ka (reference)
d
pH7.0-6.0-5.4-6.7 stepwise
pH7.0-5.4 gradient
pH7.0-5.4-6.7 gradient
CKtotal(1) / CL Total(0)
kdeg (10⁶/s)
kdeg
0
2x10⁴
3x10⁵
2x10⁵
e
pH7.0-6.0-5.4-6.7 stepwise
pH7.0-5.4 gradient
pH7.0-5.4-6.7 gradient
CL Total(x) (10⁻¹⁰M)
Height: x
f
CK total(1) - CK total(0.5) / CK total(0.5) - CK total(0)
kauto(x) (/M/s)
kmeso(x) (/M/s)
kdeg = 0
kdeg = 2x10⁴
kdeg = 3x10⁵
kdeg = 2x10⁵
g
CK total(1) / CpKLK total(0)

Supplementary Figure 5. Profiles of mathematical model parameters

a, **b** Profile of the relative catalytic activity of (**a**) KLKs ($a_{\text{KLK}}(pH(x))$) and (**b**) mesotrypsin ($a_{\text{meso}}(pH(x))$) to that at optimal pH 7.8. Data are shown as the mean ± SEM and are pooled from three experiments. Curve-fitting was based on (**a**) sixth-order and (**b**) third-order polynomial approximations. **c** pH-dependent association ($k_{\text{a}}(pH(x))$) and dissociation ($k_{\text{d}}(pH(x))$) rates between KLK/LEKTI and the KLK–LEKTI complex. Reference values were obtained from previously published data[38]. Curve-fitting was based on power approximations. **d** Relationship between k_{deg} (elimination rate constant of LEKTI via mesotrypsin) and $\frac{C_{\text{Ltotal}}(1)}{C_{\text{Ltotal}}(0)}$. Solid, dashed, and dotted lines correspond to scenarios where the total concentration of LEKTI, $C_{\text{Ltotal}}(x)$, decreased to 100% (no degradation), 50% (continuously decreased), and 0.01% (completely depleted) at x=1 (i.e. $\frac{C_{\text{Ltotal}}(1)}{C_{\text{Ltotal}}(0)} = 1, 0.5, \text{or } 0.0001$), respectively. k_{deg} values were set at 0, 2×10^4, and 3×10^5 for pH7.0_6.0_5.4_6.7 (stepwise), 0, 2×10^4, and 2×10^5 for pH7.0_5.4 (gradient), and 0, 2×10^4, and 3×10^5 for pH7.0_5.4_6.7 (gradient). **e** Profiles of $C_{\text{Ltotal}}(x)$ with different k_{deg}. The corresponding k_{deg} values represent no degradation of LEKTI (solid lines), continuously decreasing LEKTI (dashed lines), and complete depletion of LEKTI (dotted lines). **f** Relationship between [k_{deg}, k_{auto}, k_{meso}] and $\frac{C_{\text{Ktotal}}(1)-C_{\text{Ktotal}}(0.5)}{C_{\text{Ktotal}}(0.5)-C_{\text{Ktotal}}(0)}$. **g** Relationship between [k_{deg}, k_{auto}, k_{meso}] and $\frac{C_{\text{pKLK}}(1)}{C_{\text{pKLK}}(0)}$. Green solid and yellow dotted lines represent the area of k_{auto} and k_{meso} values that achieved only Eq. 21 or both Eqs. 21 and 22 (details are described in the Methods), respectively (**f** and **g**). We confirmed that [k_{deg}, k_{auto}, k_{meso}] (indicated with * in (**f**) and (**g**)) reproduced the expected increases in $C_{\text{Ktotal}}(x)$ throughout the SC ($0 < x < 1$). Those marked with a + in (**a**) and (**b**) did not. k_{auto}, rate constant of pro-KLK autoactivation; k_{meso}, rate constant of pro-KLK activation via mesotrypsin. Source data are provided as a Source Data file.

Fig. S6

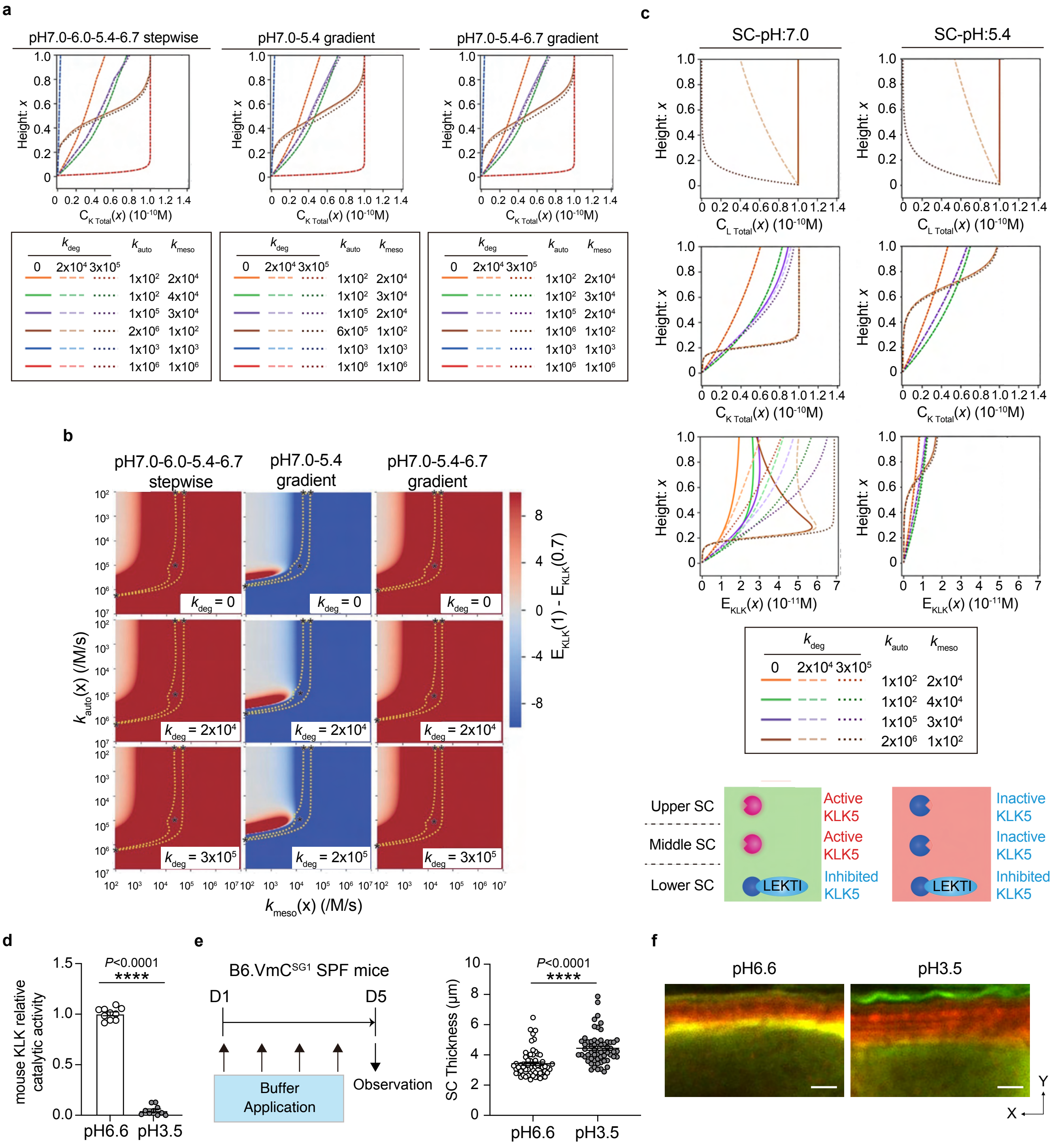

a
pH7.0-6.0-5.4-6.7 stepwise
pH7.0-5.4 gradient
pH7.0-5.4-6.7 gradient
b
c
SC-pH:7.0
SC-pH:5.4
Upper SC
Middle SC
Lower SC
Active KLK5
Inactive KLK5
Inhibited KLK5
LEKTI
d
mouse KLK relative catalytic activity
P<0.0001
pH6.6
pH3.5
e
B6.VmC SPF mice
D1
D5
Buffer Application
Observation
SC Thickness (μm)
f
pH6.6
pH3.5

Supplementary Figure 6. Profiles of $C_{\mathrm{Ltotal}}(x)$ and $C_{\mathrm{Ktotal}}(x)$ with different k_{deg} and influence of k_{deg}, k_{auto}, k_{meso} on E_{KLK}

a Influence of pH7.0_6.0_5.4_6.7 stepwise, pH7.0_5.4 gradient, and pH7.0_5.4_6.7 gradient profiles on $C_{\mathrm{Ktotal}}(x)$. **b** Relationship between [k_{deg}, k_{auto}, k_{meso}] and $E_{\mathrm{KLK}}(1.0) - E_{\mathrm{KLK}}(0.7)$. The areas surrounded by yellow dotted lines correspond to k_{auto} and k_{meso} parameter values that reproduced increases in $C_{\mathrm{Ktotal}}(x)$ throughout the SC (Figure S4F). The parameter values in those areas achieved an increase in E_{KLK} ($0 < E_{\mathrm{KLK}}(1.0) - E_{\mathrm{KLK}}(0.7)$) for pH7.0_6.0_5.4_6.7 (stepwise) and pH7.0_5.4_6.7 (gradient) but not for pH7.0_5.4 (gradient). Simulations using the parameter sets of [k_{deg}, k_{auto}, k_{meso}] marked with * in (**b**) indeed demonstrated increases in E_{KLK}. **c** Profiles of effective catalytic KLK activity ($E_{\mathrm{KLK}}(x)$), concentration of total LEKTI ($C_{\mathrm{Ltotal}}(x)$), concentration of total KLKs ($C_{\mathrm{Ktotal}}(x)$), and schematic of KLK and LEKTI in the uniform pH 5.4 (left) and pH 7.0 (right) model. The parameter sets of [k_{deg}, k_{auto}, k_{meso}] used in the simulations are those marked with * in supplementary Figure 6b. k_{deg}, elimination rate constant of LEKTI via mesotrypsin; k_{auto}, rate constant of pro-KLK autoactivation; k_{meso}, rate constant of pro-KLK activation via mesotrypsin. **d** Quantitative catalytic activity of mouse KLK at pH 6.6 and 3.5. Data are shown as mean ± SEM and have been pooled from three experiments totaling 10 biologically independent samples, each. **e** Experimental design (left). SC thickness for ear skin from B6.VmC$^{\mathrm{SG1}}$ mice treated with pH 6.6 or pH 3.5 buffer (n=60 spots from three biologically independent animals, each) (right). **f** High-magnification X-Y plane confocal images of SC-pH zones of ear skin from B6.VmC$^{\mathrm{SG1}}$ mice treated with pH 6.6 or pH 3.5 buffer (right). Data are shown as mean ± SEM and are pooled from three experiments (**e**) or are representative of at least three independent experiments (**f**). ****$p < 0.0001$; Two-sided Mann–Whitney's test (**d**, **e**). Source data are provided as a Source Data file.
