# Supplementary material for: Three stepwise pH progressions in stratum corneum for homeostatic maintenance of the skin
Source: Nat Commun. 2024 May 15;15:4062. doi: 10.1038/s41467-024-48226-z (PMC11096370; doi:10.1038/s41467-024-48226-z)
Supplement: Supplementary file 3 — Description of Additional Supplementary Files [file 41467_2024_48226_MOESM3_ESM.pdf]

<u>Description of Additional Supplementary files</u>

Supplementary Data 1: Data of mean values of single biological replicates

Supplementary Movies

Supplementary Movie 1 Representative X-Y plane and Z axis reconstruction confocal image of abdomen skin sample from VmCSG1 mouse. Video scans from the SC to the SG1 layer. The intercellular pH of the SC has three stepwise pH zones: lower-moderately acidic (yellow cytoplasm), middle-acidic (red cytoplasm), and upper-nearly neutral (green cytoplasm).

Supplementary Movie 2 Representative X-Y plane and Z axis reconstructed confocal image of ear skin sample from B6.Display-VmCSG1 mouse. Video scans from the SC to the SG1 layer. The extracellular pH of the SC has three stepwise pH zones: lower-moderately acidic (yellow cytoplasm), middle-acidic (red cytoplasm), and upper-nearly neutral (green cytoplasm).

Supplementary Movie 3 Representative X-Y plane and Z axis reconstruction confocal images of dorsal skin samples from VmCSG1 and VH148GmCSG1 mice. Video scans from the SC to the SG3 layer. To detect SG cells, keratinocyte nuclei were stained via intradermal Hoechst 33242 injection (white).

Supplementary Movie 4 Representative X-Y plane and Z axis reconstruction confocal images of ear skin samples from SPF, GF, Ex-GF B6.VmCSG1 mice, and SPF B6.VmCSG1 mice that received antibiotic treatment. Video scans from the SC to the SG1 layer.

Supplementary Movie 5 Representative X-Y plane and Z axis reconstruction confocal image of ear skin samples from B6.VmCSG1 mice inoculated with VFP-labeled S. aureus or MC903 and VFP-labeled S. aureus. Video scans from the SC to the SG1 layer. Supplementary Movie 6. Representative X-Y plane and Z axis reconstruction confocal image of ear skin samples from B6.VmCSG1 mice inoculated with MC903, VFP-labeled S. aureus, and different pH buffer (pH 6.6 and pH 10.3). Video scans from the SC to the SG2 layer. The white rectangle represents S. aureus signals entered living cell layer (below SC).
