# Supplementary material for: Three stepwise pH progressions in stratum corneum for homeostatic maintenance of the skin
Source: Nat Commun. 2024 May 15;15:4062. doi: 10.1038/s41467-024-48226-z (PMC11096370; doi:10.1038/s41467-024-48226-z)
Supplement: Supplementary file 11 — Reporting Summary [file 41467_2024_48226_MOESM11_ESM.pdf]

nature portfolio

Corresponding author(s): Masayuki Amagai

Last updated by author(s): Apr 1, 2024

Reporting Summary

Nature Portfolio wishes to improve the reproducibility of the work that we publish. This form provides structure for consistency and transparency in reporting. For further information on Nature Portfolio policies, see our Editorial Policies and the Editorial Policy Checklist.

Statistics

For all statistical analyses, confirm that the following items are present in the figure legend, table legend, main text, or Methods section.

n/a	Confirmed	
☐	☒	The exact sample size (n) for each experimental group/condition, given as a discrete number and unit of measurement
☐	☒	A statement on whether measurements were taken from distinct samples or whether the same sample was measured repeatedly
☐	☒	The statistical test(s) used AND whether they are one- or two-sided<br>*Only common tests should be described solely by name; describe more complex techniques in the Methods section.*
☐	☒	A description of all covariates tested
☐	☒	A description of any assumptions or corrections, such as tests of normality and adjustment for multiple comparisons
☐	☒	A full description of the statistical parameters including central tendency (e.g. means) or other basic estimates (e.g. regression coefficient) AND variation (e.g. standard deviation) or associated estimates of uncertainty (e.g. confidence intervals)
☐	☒	For null hypothesis testing, the test statistic (e.g. F, t, r) with confidence intervals, effect sizes, degrees of freedom and P value noted<br>*Give P values as exact values whenever suitable.*
☒	☐	For Bayesian analysis, information on the choice of priors and Markov chain Monte Carlo settings
☐	☒	For hierarchical and complex designs, identification of the appropriate level for tests and full reporting of outcomes
☒	☐	Estimates of effect sizes (e.g. Cohen's d, Pearson's r), indicating how they were calculated

Our web collection on statistics for biologists contains articles on many of the points above.

Software and code

Policy information about availability of computer code

Data collection	Inverted TCS SP8 confocal microscope (Leica Microsystems, Wetzlar, Germany); FV3000 laser scanning confocal microscope; SU8220 field emission scanning electron microscope (Hitachi High Technologies Corporation, Tokyo, Japan); iBright FL1500 Imaging system (Thermo Fisher Scientific); infinite 200Pro with Tecan i-control (Tecan Japan Co., Ltd., Kawasaki, Japan); digital micrometer (Mitutoyo, Kawasaki, Japan); PerkinElmer 2030 Manager.
Data analysis	GraphPad Prism Version 9.3.1; Fiji for Mac OS X; Photoshop Version 24.7.0; Python version 3.8.1.

For manuscripts utilizing custom algorithms or software that are central to the research but not yet described in published literature, software must be made available to editors and reviewers. We strongly encourage code deposition in a community repository (e.g. GitHub). See the Nature Portfolio guidelines for submitting code & software for further information.

Data

Policy information about availability of data

All manuscripts must include a data availability statement. This statement should provide the following information, where applicable:

- Accession codes, unique identifiers, or web links for publicly available datasets
- A description of any restrictions on data availability
- For clinical datasets or third party data, please ensure that the statement adheres to our policy

The raw data generated in this study to plot graphs and uncropped Western blots are provided in the Source Data file. The source code to reproduce the presented results is available at the online code repository: https://github.com/Tanaka-Group/stepwise_pH (DOI identifier: https://github.com/Tanaka-Group/pH_gradient).

Research involving human participants, their data, or biological material

Policy information about studies with human participants or human data. See also policy information about sex, gender (identity/presentation), and sexual orientation and race, ethnicity and racism.

Reporting on sex and gender	n/a
Reporting on race, ethnicity, or other socially relevant groupings	n/a
Population characteristics	n/a
Recruitment	n/a
Ethics oversight	n/a

Note that full information on the approval of the study protocol must also be provided in the manuscript.

Field-specific reporting

Please select the one below that is the best fit for your research. If you are not sure, read the appropriate sections before making your selection.

☒ Life sciences ☐ Behavioural & social sciences ☐ Ecological, evolutionary & environmental sciences

For a reference copy of the document with all sections, see nature.com/documents/nr-reporting-summary-flat.pdf

Life sciences study design

All studies must disclose on these points even when the disclosure is negative.

Sample size	At least 3 biologically independent replicates.
Data exclusions	All data were used.
Replication	No technical replicates were used, all studies were done with at least 3 biologically independent replicates from 3 biologically independent animals.
Randomization	No randomization of subjects was performed since this study is an observational study without predefined groups.
Blinding	No blinding was performed in data collection nor analysis since this study is an observational study without predefined groups.

Reporting for specific materials, systems and methods

We require information from authors about some types of materials, experimental systems and methods used in many studies. Here, indicate whether each material, system or method listed is relevant to your study. If you are not sure if a list item applies to your research, read the appropriate section before selecting a response.

Materials & experimental systems

n/a	Involved in the study
☐	☒ Antibodies
☒	☐ Eukaryotic cell lines
☒	☐ Palaeontology and archaeology
☐	☒ Animals and other organisms
☒	☐ Clinical data
☒	☐ Dual use research of concern
☒	☐ Plants

Methods

n/a	Involved in the study
☒	☐ ChIP-seq
☒	☐ Flow cytometry
☒	☐ MRI-based neuroimaging

Antibodies

Antibodies used	anti-filaggrin antibody (1:1000; Poly19058; BioLegend, San Diego, CA, USA); donkey anti-rabbit IgG highly cross-adsorbed secondary antibody, Alexa FluorTM 647 (1:1000; Thermo Fisher Scientific, Waltham, MA, USA); anti-GFP antibody (598; MBL Life Science, Sunnyvale, CA, USA), anti-RFP antibody (PM005; MBL Life Science), anti-cytokeratin 10 antibody (LH2; Santa Cruz Biotechnology, Dallas, TX, USA).
Validation	For western blotting, the validation of anti-filaggrin, anti-GFP, anti-RFP, and anti-cytokeratin 10 antibodies were performed with the protein obtained from D-Squame tape itself.

Animals and other research organisms

Policy information about studies involving animals; ARRIVE guidelines recommended for reporting animal research, and Sex and Gender in Research

Laboratory animals	C57BL/6 (B6); Hos:HR-1(HR); B6.Cldn1+/-; B6.SASPEGFP/+ (B6.EGFPSG1); HR.SASPEGFP/+ (EGFPSG1); B6.SASPVenusH148G-mCherry/+ (B6.VH148GmCSG1); HR.SASPVenusH148G-mCherry/+ (VH148GmCSG1); B6.SASPVenus-mCherry/+ (B6.VmCSG1); SASPVenus-mCherry/+ (VmCSG1); B6.SASPDisplay-Venus-mCherry/+ (B6.Display-VmCSG1); B6.Cldn1fl/fl K14-CreERT VH148GmCSG1; Germ-free B6.VmCSG1; 12–24 weeks-old.
Wild animals	N/A
Reporting on sex	Both male and female were included in all subsequent experiments.
Field-collected samples	N/A
Ethics oversight	All animal experiments were performed according to the guidelines approved under protocol AEY2021-018(2) and A2022-285 by the Animal Care and Use Committee of the RIKEN Yokohama Institute and Keio University Institutional Animal Care and Use Committee, respectively. All mice were anesthetized via intraperitoneal injection of a medetomidine-midazolam-butorphanol (MMB) mixture (0.75:4:5) to perform intravital imaging . In addition, all mice were euthanized under anesthesia.

Note that full information on the approval of the study protocol must also be provided in the manuscript.

Plants

Seed stocks	*Report on the source of all seed stocks or other plant material used. If applicable, state the seed stock centre and catalogue number. If plant specimens were collected from the field, describe the collection location, date and sampling procedures.*
Novel plant genotypes	*Describe the methods by which all novel plant genotypes were produced. This includes those generated by transgenic approaches, gene editing, chemical/radiation-based mutagenesis and hybridization. For transgenic lines, describe the transformation method, the number of independent lines analyzed and the generation upon which experiments were performed. For gene-edited lines, describe the editor used, the endogenous sequence targeted for editing, the targeting guide RNA sequence (if applicable) and how the editor was applied.*
Authentication	*Describe any authentication procedures for each seed stock used or novel genotype generated. Describe any experiments used to assess the effect of a mutation and, where applicable, how potential secondary effects (e.g. second site T-DNA insertions, mosiacism, off-target gene editing) were examined.*
